# The Nonlinear Relationship Between Total Bilirubin and Coronary Heart Disease: A Dose-Response Meta-Analysis

**DOI:** 10.3389/fcvm.2021.761520

**Published:** 2022-01-05

**Authors:** Chaoxiu Li, Wenying Wu, Yumeng Song, Shuang Xu, Xiaomei Wu

**Affiliations:** ^1^Department of Clinical Epidemiology and Center of Evidence-Based Medicine, The First Hospital of China Medical University, Shenyang, China; ^2^Department of Interventional Surgery, The First Hospital of China Medical University, Shenyang, China; ^3^School of Library and Medical Informatics, China Medical University, Shenyang, China

**Keywords:** bilirubin, coronary heart disease, nonlinear, dose response, meta-analysis

## Abstract

**Background:** Evidence suggests that the total bilirubin has a protective effect on coronary heart disease (CHD), but the dose-response relationship remains controversial, and there is no meta-analysis to assess the relationship.

**Methods:** As of October 1, 2021, relevant literature was selected from four databases (PubMed, Web of Science, Cochrane Library, and Embase) by using a retrieval strategy. The dose-response curve between the total bilirubin and CHD was fitted by a restricted cubic spline. Stata 12.0 was used for statistical analysis.

**Results:** A total of 170,209 (6,342 cases) participants from 7 prospective studies were analyzed in our meta-analysis. We calculated the pooled relative risks (RRs) and 95% CIs for the association between serum bilirubin level and risk of CHD using random-effects models. Compared with the first quantile, the bilirubin level in the third quantile had a protective effect on the risk of CHD (RR, 0.90; 95% CI, 0.82–0.99). The restricted cubic spline functions depicted a U-type curve relationship between bilirubin (3.42–49 μmol/L) and CHD (*P*_linear_ < 0.001). When the bilirubin level was in the range of 3.42–13μmol/L, the protective effect of bilirubin on CHD was enhanced with increasing bilirubin levels. When the bilirubin level exceeded 13μmol/L, the protective effect of bilirubin weakened, and a dangerous effect gradually appeared with further increases in bilirubin levels.

**Conclusions:** Compared with a low bilirubin level, a high bilirubin level has a protective effect on the risk of CHD, and there was a U-shaped dose-response relationship between them.

## Introduction

Coronary heart disease (CHD) accounts for one-third to one-half of cardiovascular diseases and is the most common heart disease (1). According to WHO estimates, the number of deaths from cardiovascular disease has increased by more than 2 million since 2000 to nearly 9 million in 2019 (2). The increase in cardiovascular disease deaths was due to an increase in the prevalence of CHD (3). Bilirubin is formed from biliverdin through heme catabolism and biliverdin reductase activity (4). Traditionally, bilirubin is considered toxic and can cause irreversible damage to the nervous system (5). In the clinic, bilirubin concentration is used as a marker for the diagnosis of some liver diseases (6). However, there is evidence that bilirubin has antioxidant activity, and its antioxidant effect is stronger than that of bilirubin α-tocopherol (7). It plays an important role in the occurrence and development of oxidative stress diseases. Recent studies have shown that bilirubin has a certain clinical value in the diagnosis and prognosis of stroke (8), diabetes (9), peripheral arterial disease (10), Parkinson's disease (11), and heart failure (12), so it may be used as a new marker for cardiovascular and cerebrovascular diseases in clinical practice.

At present, the relationship between bilirubin and the risk of CHD has attracted increasing attention from scholars. However, the conclusions of these studies are not consistent. Some studies reported that bilirubin was not associated with the occurrence of CHD (13–15); some studies believe that there is a negative linear relationship between them, and with the increase of bilirubin level, the risk of CHD decreases accordingly (16); interestingly, other studies believe that there is a nonlinear relationship between them, such as L-type (17) and U-type (18–20). Stender et al. (21) conducted a meta-analysis of the relationship between hereditary elevated bilirubin levels and the risk of CHD and found no evidence of the association between bilirubin and CHD. Subsequently, a prospective meta-analysis of the relationship between bilirubin and cardiovascular disease reported consistent results (22). Their study only considered whether bilirubin was related to CHD but did not evaluate the relationship between bilirubin exposure level and CHD.

Considering that bilirubin may have certain potential value in the occurrence and development of CHD and no consistent conclusion has been reached on the relationship between the two, it is necessary to explore bilirubin and CHD. Based on published epidemiological studies, we calculated the relative risk of CHD by dose-response meta-analysis, and a dose-response curve was drawn by restrictive cubic spline function to obtain more accurate and intuitive evidence of the dose-response relationship between them.

## Methods

This systematic review was conducted according to the guidelines of the Preferred Reporting Items for Systematic Reviews and Meta-Analyses (PRISMA) (23) (Supplementary Table 1). Since it is an observational study, the principle of Population, Intervention, Comparator, and Outcomes (PICO) is not suitable, and we adopt the concept of population exposure comparator outcome (PECO) as pillars of the question (24):

P: People of any age,
E: Third quantile of bilirubin level,
C: First quantile of bilirubin level,
O: CHD.

### Search Strategy

An extensive search strategy was carried out in four databases (PubMed, Web of Science, Cochrane Library, and Embase) for studies published before October 1, 2021. The following search keywords were used: “bilirubin and CHD,” “bilirubin and cardiovascular diseases,” and “bilirubin and ischemia heart disease.” Relevant MeSH terms were searched in combination to identify relevant studies. Refer to Table 1 for the detailed method used to search PubMed. The strategies for the other databases were similar but adapted where necessary. We also searched references to reviews with related or similar themes for additional research (21, 22, 25–27).

**Table 1:** Strategies for the database search.

**#1**	**“Bilirubin”[Mesh]**
#2	(((((((((((((((Bilirubin IX alpha[Text Word]) OR (Bilirubin, (4E)-Isomer[Text Word])) OR (Bilirubin, (4E,15E)-Isomer[Text Word])) OR (Hematoidin[Text Word])) OR (Bilirubin, Disodium Salt[Text Word])) OR (Disodium Salt Bilirubin[Text Word])) OR (Bilirubin, Monosodium Salt[Text Word])) OR (Monosodium Salt Bilirubin[Text Word])) OR (delta-Bilirubin[Text Word])) OR (delta Bilirubin[Text Word])) OR (Bilirubin, (15E)-Isomer[Text Word])) OR (Bilirubin, Calcium Salt[Text Word])) OR (Calcium Salt Bilirubin[Text Word])) OR (Salt Bilirubin, Calcium[Text Word])) OR (Calcium Bilirubinate[Text Word])) OR (Bilirubinate, Calcium[Text Word])
#3	#1 OR #2
#4	(((“Cardiovascular Diseases”[Mesh]) OR “Coronary Disease”[Mesh])) OR “Myocardial Ischemia”[Mesh]
#5	((((((((((((((((((((Cardiovascular Disease[Text Word]) OR (Disease, Cardiovascular[Text Word])) OR (Diseases, Cardiovascular[Text Word])) OR (Coronary Diseases[Text Word])) OR (Disease, Coronary[Text Word])) OR (Diseases, Coronary[Text Word])) OR (Coronary Heart Disease[Text Word])) OR (Coronary Heart Diseases[Text Word])) OR (Disease, Coronary Heart[Text Word])) OR (Diseases, Coronary Heart[Text Word])) OR (Heart Disease, Coronary[Text Word])) OR (Heart Diseases, Coronar[Text Word])) OR (Ischemia, Myocardial[Text Word])) OR (Ischemias, Myocardial[Text Word])) OR (Myocardial Ischemias[Text Word])) OR (Ischemic Heart Disease[Text Word])) OR (Heart Disease, Ischemic[Text Word])) OR (Disease, Ischemic Heart[Text Word])) OR (Diseases, Ischemic Heart[Text Word])) OR (Heart Diseases, Ischemic[Text Word])) OR (Ischemic Heart Diseases[Text Word])
#6	#4 OR #5
#7	#3 OR #6

### Inclusion and Exclusion Criteria

According to the inclusion criteria, the possibly relevant articles that had been collected were independently assessed by two authors (CXL and YMS). The third author (XMW) resolved any disagreements by discussion or consultation.

Articles were included if they fulfilled all of the following criteria: (1) the article reported the risk estimate and 95% CI of the relationship between bilirubin and CHD; (2) the design of the study was prospective; (3) Provided risk estimates at different bilirubin exposure levels, or provided corresponding data to calculate them; (4) Provided sample sizes at different bilirubin exposure levels, or provided corresponding data to calculate them; (5) the sample source of the article was human; (6) if multiple articles were published from the same/similar population, we included data from the study with the largest sample size and/or the latest or highest-quality result. Reviews, case reports, lab experiments, duplicate publications, and articles without full text or available data were excluded.

### Risk of Bias and Methodological Quality

The risk of bias and methodological quality of the included studies were independently assessed by two reviewers (CXL and WYW) using the Newcastle Ottawa scale (NOS), with an asterisk assigned to each article. A study can get up to one star for each numbered item in the selection and outcome categories and up to two stars for comparability. One asterisk equals one score, and the quality of the studies was graded as poor (fewer than four scores), fair (four to six scores), and good (seven or more scores) (28, 29).

### Data Extraction

Data extraction was managed in Microsoft Excel. The following information was independently extracted from the included articles by two authors (CXL and WYW): author, year of publication, country, study design, sample source, follow-up period, sample size (case/total), effect value, adjusted risk factors, baseline characteristics [age, body mass index (BMI), smokers, alcohol], diseases (hypertension, diabetes, hyperlipidemia, and liver disease), drug, and biochemical indicators of the subjects. For studies that presented several estimates adjusted for different numbers of potential confounders, the estimate that adjusted for the maximum number of potential confounders was selected for analysis.

In addition, we also extracted the following information for the dose-response meta-analysis: (1) the risk estimates and their corresponding 95% CIs in each group; (2) the median or mean of total bilirubin concentration in each group; and (3) the number of cases and total number in each group.

### Statistical Analysis

In our meta-analysis, pooled risk ratios (RRs) and 95% CIs were used to analyze the association between serum bilirubin and the risk of CHD. The result of the pooled estimate was generated using the adjusted odds ratio (OR), relative risk (RR), hazard ratio (HR), and a baseline measure of total bilirubin exposure. In the original research, the effect value (OR/RR/HR) was reported with different distributions of bilirubin levels (such as per tertiles, quartiles, quintiles in bilirubin levels). To enable a consistent approach to the meta-analysis and enhance the interpretation of findings, ORs were converted into RRs by the formula, and HRs were regarded as approximate to RRs (30, 31). At the same time, as reported in previous studies, the results were transformed into RR between the upper tertiles and lower tertiles (32). The chi-square-based Q-test was used to assess the heterogeneity among the individual studies (33). Heterogeneity was quantified based on I^2^, which ranged from 0 to 100% (I^2^ = 0–25%, no heterogeneity; I^2^ = 25–50%, low heterogeneity; I^2^ = 50–75%, moderate heterogeneity; I^2^ = 75–100%, high heterogeneity). Considering the underlying clinical and methodological heterogeneity (e.g., baseline characteristics of the patients, adjustment for confounders, and follow-up duration), a random-effects model was used as the primary approach to combine results across studies (34). The possible sources of heterogeneity were explored by subgroup analysis. Egger tests were used to evaluate the possibility of publication bias (35). The sensitivity analysis was performed by excluding one study at a time to evaluate whether the results could have been affected by a single study (36). A two-sided value of *P* < 0.05 in the statistical process was considered statistically significant (37).

We used the two-stage generalized least squares method to estimate the relative risk of the original research report and established a restrictive cubic spline curve with five nodes (5, 25, 50, 75, and 95) to fit the dose-response relationship between bilirubin and CHD (38–40). To ensure unit consistency in all included studies, we converted mg/dL to μmol/L by multiplying by 17.1. If the article reported exposure category by a range, the midpoint was calculated by averaging the lower and upper bounds; if the lowest boundary was open-ended, the midpoint was set at half of the upper boundary; and when the upper boundary for the highest group was not provided, the midpoint was set at 1.5 times the lower boundary.

The dose-response meta-analysis was conducted using the Stata software package (Version 12.0; Stata Crop, College Station, TX, USA).

## Results

### Literature Search

**Figure 1:**
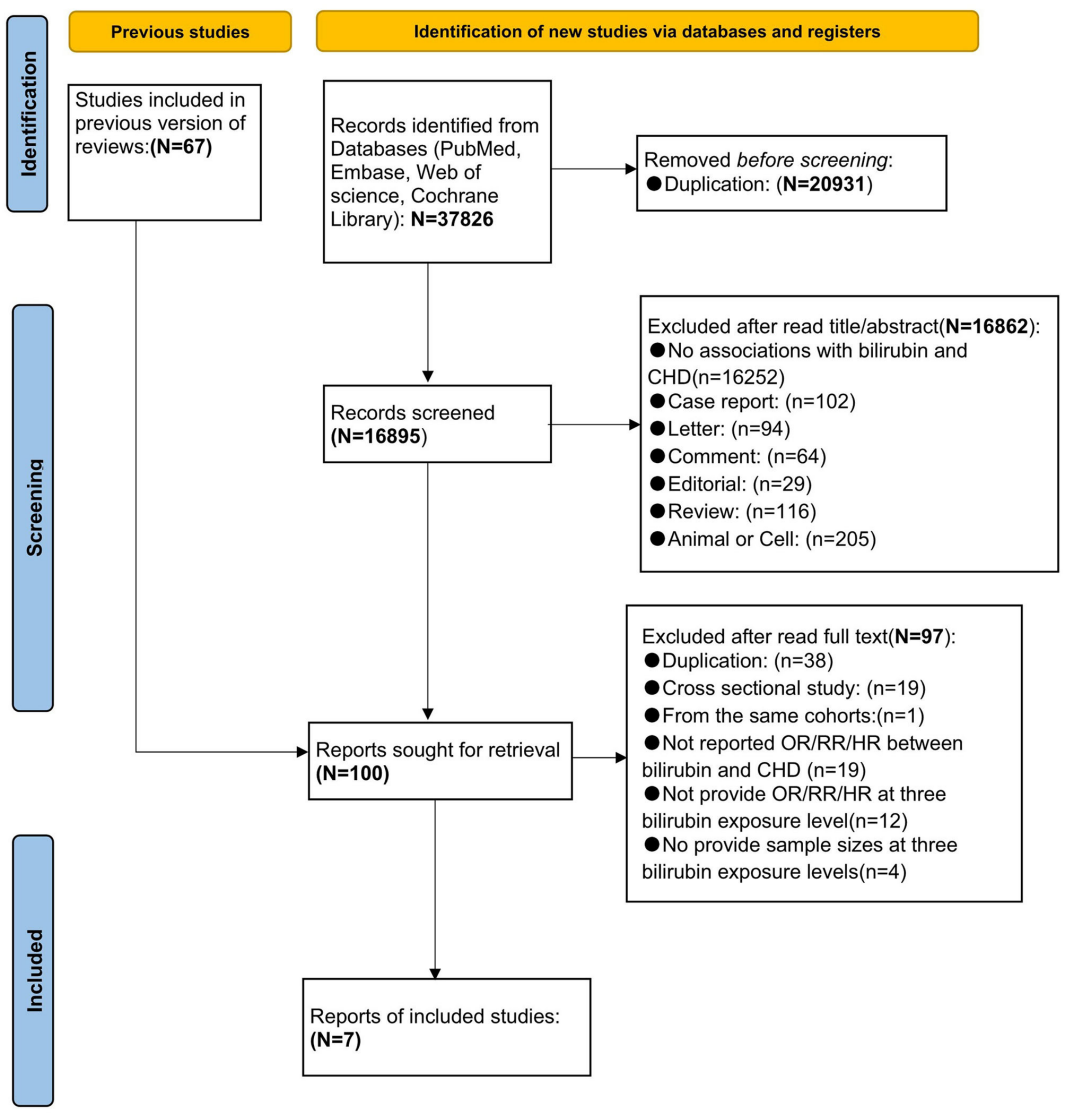
Preferred Reporting Items for Systematic Reviews and Meta-Analyses (PRISMA) flow chart of literature retrieval and selection.

A total of 37,826 potential studies were retrieved. A total of 20,931 articles were excluded according to automation tools. The remaining literature was imported into EndNote X9 (Clarivate Analytics) for further screening. According to the analysis of titles and abstracts, 16,862 articles were excluded and 33 potential articles were included. In addition, 67 articles were included from the reference list of previous reviews. After reading the full text and detailed screening, 93 articles were excluded for various reasons. Finally, 7 prospective studies (16, 18, 20, 21, 41–43) were included in the meta-analysis. The PRISMA flowchart of literature retrieval and selection is presented in Figure 1.

### Characteristics of Studies and Risk of Bias

Table 2 shows the details of the included studies in our meta-analysis. All the effect values in the included studies were adjusted for the maximum number of potential confounders. All of the studies were based on community populations. The publication year was from 1996 to 2018. The follow-up period ranged from 5 to 21.9 years. The included studies involved three regions, including Europe (20, 21, 42), Asia (16, 18) and America (41, 43).

**Table 2:** Characteristics of articles included in the meta-analysis.

**ID**	**Author**	**year**	**Country**	**sample source**	**Follow up period**	**Subgroup**	**Case/Total**	**Effect value**	**Adjusted Basic indicators**	**Adjusted Biochemical**	**Adjusted Disease**	**Adjust-Relevant treatment**
1	Breimer	1996	British	Population base	11.5 years	measured before 16 o'clock	470/4916	RR:0.99(0.73–1.34)	age, sex, BMI, smoking, alcohol, SBP, social class, physical activity,	TC, HDL, FEV1, blood glucose, serum albumin	diabetes, preexisting IHD	antihypertensive
						measured after 16 o'clock	267/2769	RR:1.20(0.80–1.80)	age, sex, BMI, smoking, alcohol, SBP, social class, physical activity,	TC, HDL, FEV1, blood glucose, serum albumin	diabetes, preexisting IHD	antihypertensive
2	Luc	2001	American	Population base	21.9 ± 4.3 years	men	196/1983	HR:0.75(0.49–1.14)	sex, age, smoking, alcohol	TC	hypertension, diabetes	/
						women	61/2293	HR:0.53(0.18–1.52)	Sex, age, smoking, alcohol	TC	hypertension, diabetes	/
3	Stender	2013	Denmark	Population base	7.5 years	/	1583/43708	HR:0.93(0.82–1.06)	age, sex, smoking, alcohol, physical activity,	HDL-c, LDL-c, TG, CRP	hypertension, diabetes	use of lipid-lowering drugs
4	Jorgens	2014	British		6 years	/	355/9742	HR:0.73(0.545–0.979)	age, sex	/	overweight and obese	sibutramine/ placebo,
5	Lai	2018	China	Population base	5 years	/	1204/12097	HR:1.01(0.84–1.2)	age, sex, BMI, smoking, alcohol, education levels, physical activity,	ALT, AST, ALP	hypertension, diabetes, hyperlipidemia, history of CHD,	/
6	Suh	2018	Korea	Population base	8.1 years	/	444/8844	HR:0.769(0.593–0.996)	age, sex, BMI, SB,	LDL, HSCRP, HbA1c,	/	/
7	Marconi	2018	American	Population base	5.7 years	/	1762/83857	HR:0.81(0.7–0.93)	age, sex, smoking, alcohol, SBP, race-ethnicity	/	diabetes, HIV, hepatitis C, liver fibrosis measured by FIB-4	/

*IHD, ischemic heart disease; MI, myocardial infarction; CHD, coronary heart disease; RR, relative risk; HR, hazard risk; SBP, systolic blood pressure; TC, total cholesterol; HDL-c, high-density lipoprotein cholesterol; FEV1,forced expiratory volume in 1; LDL-c, low-density lipoprotein cholesterol; TG, triglycerides; CPR, C-reaction protein; ALT, alanine aminotransferase; AST, aspartate aminotransferase; ALP, alkaline phosphatase; BMI, body mass index; HIV, human immunodeficiency virus; FIB-4,fibrinogen; HbA1c, hemoglobin A1c*.

Table 3 shows the baseline characteristics and biochemical indicators of the subjects in each study. The total sample size is 170,209 (age: 27.5–70 years). The percentage of men ranged from 45 to 100%. The percentages of smoking and alcohol were 67 and 70%, respectively. The percentages of hypertension patients, diabetic patients, hyperlipidemia patients, and liver disease were 60, 70, 80, and 22.95%, respectively. The biochemical indicators included AST, ALT, TC, TG, LDL, and HDL. The biochemical indicators of the subjects in all the included studies are shown in detail in Table 3.

**Table 3:** Characteristics of subjects included in the study.

**Author**	**Sample size**	**Male (%)**	**Age (year)**	**BMI (kg/m2)**	**Smoker (%)**	**Alcohol (%)**	**Hypertension (%)**	**Diabetes (%)**	**Hyperlipidemia (%)**	**Liver disease (%)**	**Drug (%)**	**bilirubin level (μmol/L)**	**TC (mmol/L)**	**TG (mmol/L)**	**HDL (mmol/L)**	**LDL (mmol/L)**	**AST (U/l)**	**ALT (U/l)**
Breimer	7685	100%	/	/	/	/	/	/	/	/	/	8.47	/	/	/	/	/	/
Luc	4276	46%	37.42	/	/	/	19%	2%	/	0%	/	12.60	5.09	/	/	/	/	/
Stender	43708	59%	54.65	25.35	22%	42%	54%	3%	7%	/	/	10.05	5.59	1.51	1.64	3.22	/	/
Jorgens	9742	57%	62.91	34.46	42%	/	/	/	/	5%	66%	11.02	/	/	46.23	107.15	23.25	26.03
Lai	12097	45%	62.7	24.26	19%	23%	48%	16%	49%	/	/	13.75	/	/	/	/	23.15	20.37
Suh	8844	47%	52.3	24.56	25%	/	31%	/	18%	/	/	12.83	5.01	1.79	1.12	3.08	28.19	26.82
Marconi	83857	97%	48.56	/	52%	27%	25%	14%	/	46%	17%	10.56	/	/	/	/	/	/

*TC, total cholesterol; HDL, high-density lipoprotein cholesterol; LDL, low-density lipoprotein cholesterol; TG, triglycerides; ALT, alanine aminotransferase; AST, aspartate aminotransferase; ALP, alkaline phosphatase; BMI, body mass index*.

*Age, BMI, bilirubin, TG, TC, HDL, LDL, AST, and ALT are expressed as means; smoker, alcohol, hypertension, diabetes, hyperlipidemia, liver disease, and drug are expressed as percentages*.

Table 4 shows the quality scores of the included studies based on the NOS assessment tool. All 7 articles were assessed as good.

**Table 4:** Newcastle-Ottawa Scale (NOS) scores of the studies included in the meta-analysis.

**Newcastle-Ottawa Quality Assessment Scale**									
	**Selection**				**Comparability**	**outcome**			**Quality scores ^**a**^**
	Is the case definition adequate/ Representativeness of the exposed cohort	Representativeness of the cases/ Selection of the non-exposed cohort	Selection of Controls/ Ascertainment of exposure to implants	Definition of Controls/ Demonstration that outcome of interest was not present at start of study	Comparability of cases and controls on the basis of the design or analysis/ Comparability of cohorts on the basis of the design or analysis	Ascertainment of exposure/ Assessment of outcome	Same method of ascertainment for cases and controls/ Was follow up long enough for outcomes to occur	Non-Response Rate/ Adequacy of follow up of cohorts	
Breimer	⋆	⋆	⋆	⋆	⋆⋆	⋆	⋆	⋆	9
Luc	⋆	⋆	⋆	⋆	⋆⋆	⋆	⋆	⋆	9
Stender	⋆	⋆	⋆	⋆	⋆⋆	⋆	⋆	⋆	9
Jorgens	⋆	⋆	⋆	⋆	⋆⋆	⋆	⋆	⋆	9
Lai	⋆	⋆	⋆	⋆	⋆⋆	⋆	⋆	⋆	9
Suh	⋆	⋆	⋆	⋆	⋆⋆	⋆	⋆	⋆	9
Marconi	⋆	⋆	⋆	⋆	⋆⋆	⋆	⋆	⋆	9

*a, One asterisk equals one score*.

### Correlation Between Total Bilirubin and CHD

**Figure 2:**
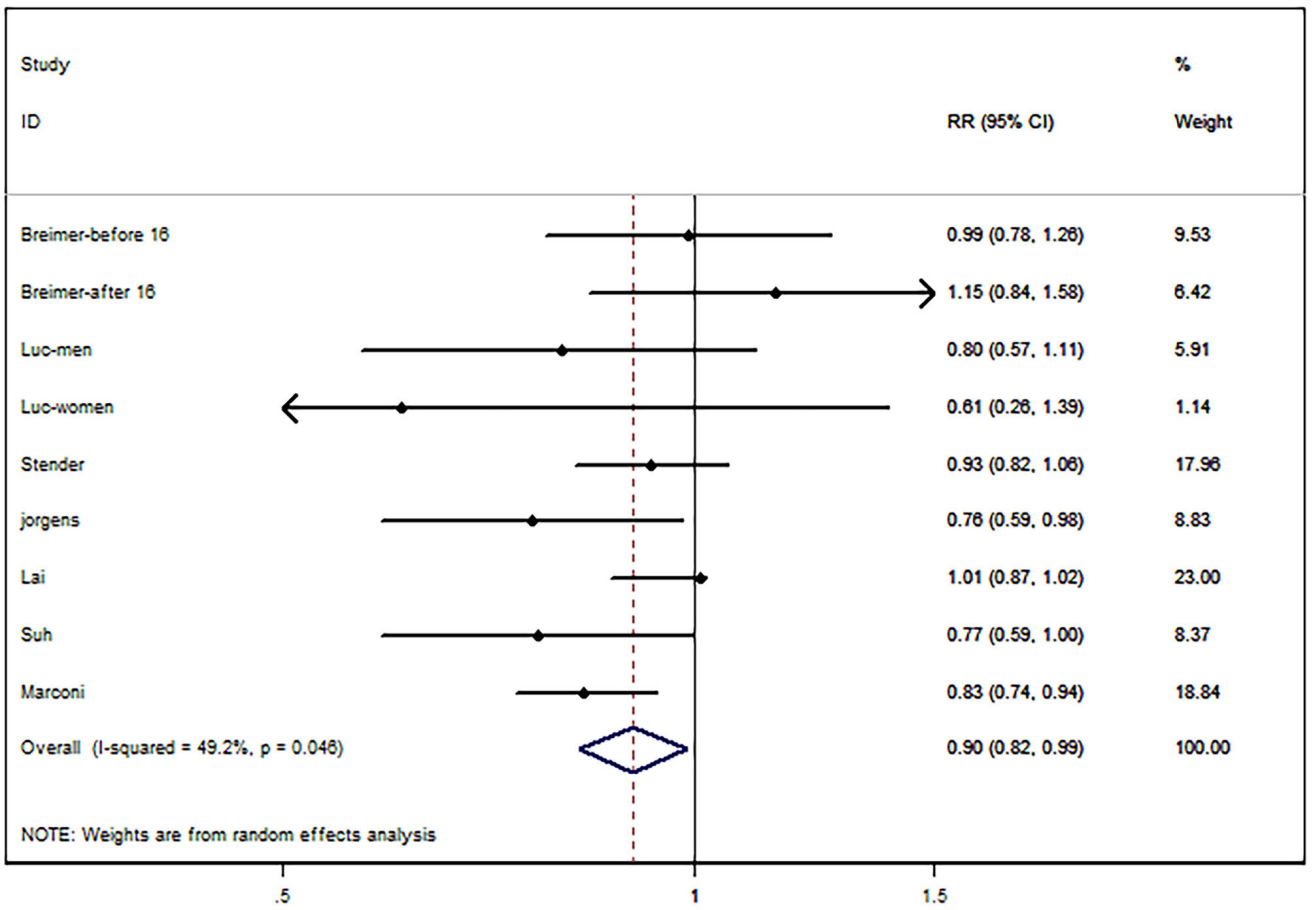
Forest map of bilirubin and risk of coronary heart disease (CHD).

We extracted 9 sets of statistical data from 7 articles, and 1 study reported the relationship between bilirubin and CHD by sex (43); in another study, the relationship between bilirubin and CHD was reported by two different populations, which were divided according to the time of bilirubin measurement (before and after 16 o'clock) (20). There was low heterogeneity in 7 studies (I^2^ = 49.2%, *P* = 0.046), and a random effect model was used for analysis. Compared with the first quantile, the bilirubin level in the third quantile had a protective effect on the risk of CHD (RR, 0.90; 95% CI, 0.82–0.99; *P* = 0.027; Figure 2.

**Figure 3:**
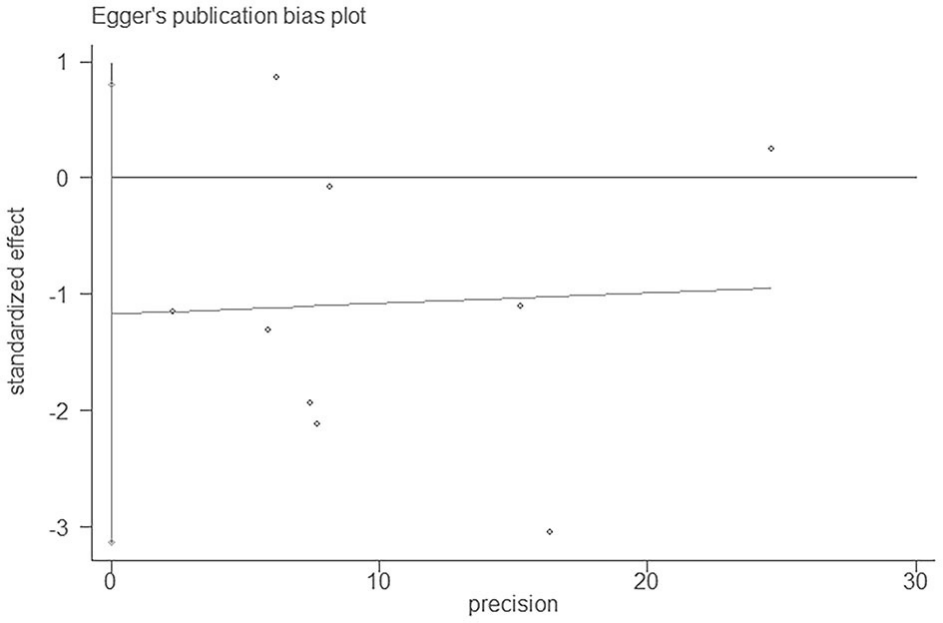
Egger's publication bias plot of bilirubin and CHD.

Subgroup analysis was performed according to sample size (subject <15,000, ≥15,000), which was consistent with the results of the holistic analysis (Table 5). Egger's test (*P* = 0.202) suggested no publication bias (Figure 3). For sensitivity analysis, the pooled RRs ranged from 0.89 (95% CI:0.81–1) to 0.92 (95% CI:0.83–1.02) (Table 6).

**Table 5:** Subgroup analysis of the relationship between total bilirubin and risk of coronary heart disease (CHD).

	**Number**	**RR (95%CI)**	**I^**2**^**	***p*** **for I^2^**
Sample size				
<15000	7	0.96(0.91–1.03)	45.8%	0.086
≥15000	2	0.88(0.78–0.98)	38.1%	0.304

*RR, relative risk; CI, confidence interval*.

**Table 6:** Sensitivity analysis of bilirubin and CHD.

**Study ommited**	**RR**	**95%CI**	
		low	up
Total	0.9	0.82	0.99
Bremier-before 16	0.89	0.81	0.99
Bremier-after 16	0.89	0.81	0.97
Luc-men	0.91	0.83	1
Luc-women	0.91	0.83	0.99
Stend	0.89	0.8	1
Jorgens	0.92	0.84	1.01
Lai	0.87	0.8	0.95
Suh	0.92	0.83	1.01
Marco	0.92	0.84	1.02

*RR, relative risk; CI, confidence interval*.

### Dose-Response Analysis for Bilirubin and CHD

**Figure 4:**
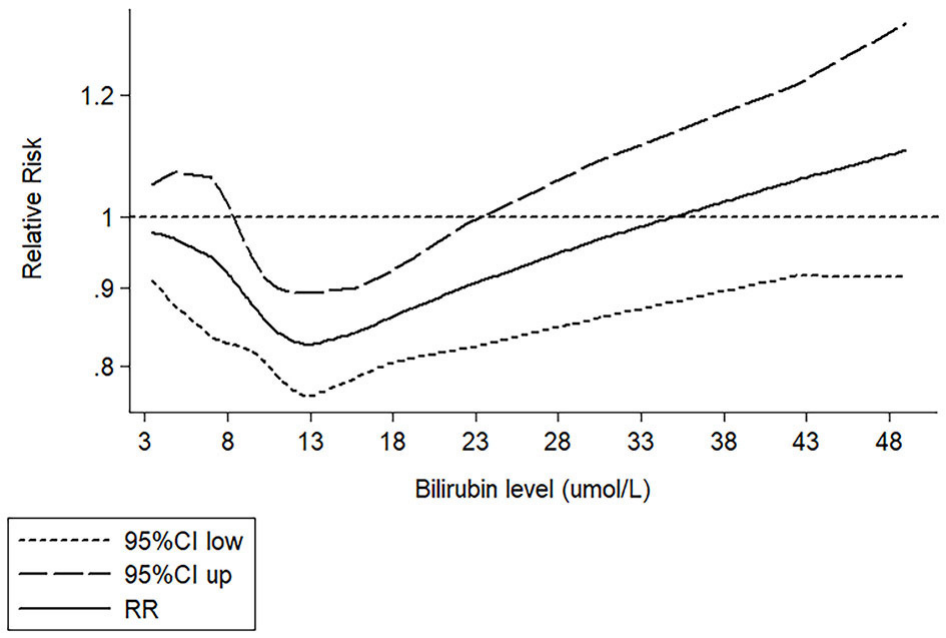
Restrictive cubic spline fitting curve between bilirubin and CHD.

We extracted 9 sets of data from 7 studies for dose-response meta-analysis (16, 18, 20, 21, 41–43) (Supplementary Table 2) The dose-effect relationship between bilirubin (3.42–49 μmol/L) and CHD was nonlinear (p-_linear_ <0.001) (Figure 4). When the bilirubin level was in the range of 3.42–13 μmol/L, the protective effect of bilirubin on CHD was enhanced. When the bilirubin level exceeded 13 μmol/L, the protective effect of bilirubin weakened, and dangerous effects gradually appeared with increasing bilirubin levels.

## Discussion

This was the first study to assess the relationship between bilirubin and CHD based on a dose-response meta-analysis. Our results showed that compared with the first quantile, the bilirubin level in the third quantile has a protective effect on the risk of CHD; at the same time, dose-response meta-analysis showed that the protective effect was U-type.

Previous studies have reported a nonlinear relationship between bilirubin and CHD, and our study is consistent with these findings. A cross-sectional study in Japan reported a nonlinear relationship between bilirubin and CHD (44). A prospective study of middle-aged people in Britain reported consistent results, and their results showed that the nonlinear relationship may be U-shaped (20). However, these conclusions are limited to male subjects. In another prospective study including Chinese elderly individuals (mean age was 62.7 years; 45.2% men), Lai et al. (18) reported a U-shaped relationship between them. Due to different research methods and subject populations, the conclusions of these studies may have some limitations, but they showed that the protective effect of bilirubin on CHD is not a simple linear relationship. The U-shaped relationship between bilirubin and disease means that when the bilirubin level is approximately 12–16 μmol/L (18, 20, 45, 46), the protective effect of bilirubin on disease will no longer be enhanced. When the bilirubin level exceeds this range, the protective effect will weaken, and a dangerous effect will begin to appear. Other studies have reported an absence of any such association, and the inconsistency of this conclusion can be attributed to the bilirubin exposure level of subjects included in the study not being high enough to detect an effect of bilirubin on CHD (15–17). For example, the results of Kim et al. (15) and Suh et al. (16) showed that there was a negative linear relationship between bilirubin and CHD. In their study, the fourth quartile of bilirubin levels was only 9.5 and 10.77 μmol/L. Therefore, the linear relationship of negative correlation they found may only be part of the U-type relationship. A similar dose-response relationship was also found between bilirubin and other diseases, such as stroke and diabetic retinopathy (DR). Liu et al. (45) reported that the protective relationship between bilirubin and stroke is nonlinear, and the risk of first ischemic stroke was the lowest when the bilirubin level was 17.0 μmol/L. A nonlinear correlation relationship was also reported between bilirubin and diabetic retinopathy (DR). The protective effect of bilirubin level on DR was the strongest in the range of 12–13.8μmol/L (46).

The change in bilirubin level plays an important role in the occurrence and development of CHD. On one hand, bilirubin can eliminate reactive oxygen species produced in the process of oxidative stress, inhibit the aggregation of inflammatory cells, improve the bioavailability of Nitric Oxide (NO), and play a protective role in the heart (47, 48). However, an increase in bilirubin exceeding the physiological range may indicate potential liver injury, and ALP, ALT, and GGT released by liver injury are related to an increased risk of CHD (49, 50). On the other hand, we should pay attention to the fact that bilirubin is also cytotoxic. It has been confirmed in animal experiments that when the accumulation of bilirubin in cells reaches a certain threshold, oxidation will change into cytotoxicity (51). Therefore, the U-type relationship between bilirubin and CHD may be a combination of antioxidant, hepatotoxicity, or cytotoxicity.

Our study has some advantages. Firstly, we established a restrictive cubic spline to fit the dose-response relationship between bilirubin and CHD by pooling original research and obtaining a more intuitive curved shape. Secondly, our dose-response meta-analysis included 7 prospective studies, a large sample size (170,209), and a wide range of bilirubin levels (3.42–49μmol/L), providing sufficient statistical validity for the dose-response relationship model. Our study also has some limitations. Firstly, in a longitudinal analysis, biochemical indicators including bilirubin were collected from baseline measurement, which are sensitive to changes during the follow-up. It may fail to (at least partly) show a robust link between bilirubin and CHD. Secondly, bilirubin levels are disturbed by many endogenous and environmental factors, we had no access to individual participants' data and potential confounders could not be ruled out. Under confounding factors, there may be no reliable connection between bilirubin and CHD. Gajdos et al. (52) and Mahaba et al. (53) found a modest link of bilirubin to CHD. However, after adjusting for risk factors, they were no longer statistically significant. Similarly, in a retrospective cohort of patients with HIV, bilirubin was associated with ischemic events. After the addition of related treatment to the Cox model, bilirubin was no longer significantly associated with ischemic events (54). Their results suggested that the correlation between bilirubin and CHD may be related to uncontrolled confounding factors. Considering the influence of potential factors, we prioritize OR/RR/HR adjusted by most factors when collecting and extracting effect values. It is hoped that the combined value can present objective results after controlling the bias factors as much as possible. In future research to explore the relationship between bilirubin and CHD, bilirubin-related factors should also be considered when adjusting the risk factors for cardiovascular disease.

## Conclusion

Compared with a low bilirubin level, a high bilirubin level has a protective effect on the risk of CHD, and there was a U-shaped dose-response relationship between them.

## Data Availability Statement

The original contributions presented in the study are included in the article/Supplementary Material, further inquiries can be directed to the corresponding author.

## Author Contributions

XW and WW designed the whole research. CL, SX, and YS conducted the data collection. CL, WW, and XW analyzed the data. CL and WW wrote the manuscript. All authors discussed the relevant results, read and approved the final manuscript.

## Conflict of Interest

The authors declare that the research was conducted in the absence of any commercial or financial relationships that could be construed as a potential conflict of interest.

## Publisher's Note

All claims expressed in this article are solely those of the authors and do not necessarily represent those of their affiliated organizations, or those of the publisher, the editors and the reviewers. Any product that may be evaluated in this article, or claim that may be made by its manufacturer, is not guaranteed or endorsed by the publisher.
