# Supplementary material for: The Nonlinear Relationship Between Total Bilirubin and Coronary Heart Disease: A Dose-Response Meta-Analysis
Source: Front Cardiovasc Med. 2022 Jan 5;8:761520. doi: 10.3389/fcvm.2021.761520 (PMC8766987; doi:10.3389/fcvm.2021.761520)
Supplement: Supplementary file 1 [file Data_Sheet_1.PDF]

Supplementary Material

Supplementary Tables

Supplementary Table S1 PRISMA checklist.

Supplementary Table S2 Data for dose response Meta-analysis.

Supplementary Table S1 PRISMA checklist.

Section and Topic	Item #	Checklist item	Location where item is reported
**TITLE**			
Title	1	Identify the report as a systematic review.	Title
**ABSTRACT**			
Abstract	2	See the PRISMA 2020 for Abstracts checklist.	Supplement Table S1
**INTRODUCTION**			
Rationale	3	Describe the rationale for the review in the context of existing knowledge.	Line 37-59
Objectives	4	Provide an explicit statement of the objective(s) or question(s) the review addresses.	Line 60-65
**METHODS**			
Eligibility criteria	5	Specify the inclusion and exclusion criteria for the review and how studies were grouped for the syntheses.	Line 84-95
Information sources	6	Specify all databases, registers, websites, organisations, reference lists and other sources searched or consulted to identify studies. Specify the date when each source was last searched or consulted.	Line 76-82
Search strategy	7	Present the full search strategies for all databases, registers and websites, including any filters and limits used.	Table 1
Selection process	8	Specify the methods used to decide whether a study met the inclusion criteria of the review, including how many reviewers screened each record and each report retrieved, whether they worked independently, and if applicable, details of automation tools used in the process.	Line 84-86
Data collection process	9	Specify the methods used to collect data from reports, including how many reviewers collected data from each report, whether they worked independently, any processes for obtaining or confirming data from study investigators, and if applicable, details of automation tools used in the process.	Line 104-113
Data items	10a	List and define all outcomes for which data were sought. Specify whether all results that were compatible with each outcome domain in each study were sought (e.g. for all measures, time points, analyses), and if not, the methods used to decide which results to collect.	Line 105-110
	10b	List and define all other variables for which data were sought (e.g. participant and intervention characteristics, funding sources). Describe any assumptions made about any missing or unclear information.	Line 105-110
Study risk of bias assessment	11	Specify the methods used to assess risk of bias in the included studies, including details of the tool(s) used, how many reviewers assessed each study and whether they worked independently, and if applicable, details of automation tools used in the process.	Line 97-102
Effect measures	12	Specify for each outcome the effect measure(s) (e.g. risk ratio, mean difference) used in the synthesis or presentation of results.	Line 115-116
Synthesis methods	13a	Describe the processes used to decide which studies were eligible for each synthesis (e.g. tabulating the study intervention characteristics and comparing against the planned groups for each synthesis (item #5)).	Line 104
	13b	Describe any methods required to prepare the data for presentation or synthesis, such as handling of missing summary statistics, or data conversions.	Line 116-123
	13c	Describe any methods used to tabulate or visually display results of individual studies and syntheses.	Line 141-142

Section and Topic	Item #	Checklist item	Location where item is reported
	13d	Describe any methods used to synthesize results and provide a rationale for the choice(s). If meta-analysis was performed, describe the model(s), method(s) to identify the presence and extent of statistical heterogeneity, and software package(s) used.	Line 123-126
	13e	Describe any methods used to explore possible causes of heterogeneity among study results (e.g. subgroup analysis, meta-regression).	Line 126-129
	13f	Describe any sensitivity analyses conducted to assess robustness of the synthesized results.	Line 130-131
Reporting bias assessment	14	Describe any methods used to assess risk of bias due to missing results in a synthesis (arising from reporting biases).	Line 129-130
Certainty assessment	15	Describe any methods used to assess certainty (or confidence) in the body of evidence for an outcome.	Line 131-132
**RESULTS**			
Study selection	16a	Describe the results of the search and selection process, from the number of records identified in the search to the number of studies included in the review, ideally using a flow diagram.	Line 145-151<br>Figure 1
	16b	Cite studies that might appear to meet the inclusion criteria, but which were excluded, and explain why they were excluded.	Figure 1
Study characteristics	17	Cite each included study and present its characteristics.	Table 2 and table 3
Risk of bias in studies	18	Present assessments of risk of bias for each included study.	Table 4
Results of individual studies	19	For all outcomes, present, for each study: (a) summary statistics for each group (where appropriate) and (b) an effect estimate and its precision (e.g. confidence/credible interval), ideally using structured tables or plots.	Table 2
Results of syntheses	20a	For each synthesis, briefly summarise the characteristics and risk of bias among contributing studies.	Figure 2
	20b	Present results of all statistical syntheses conducted. If meta-analysis was done, present for each the summary estimate and its precision (e.g. confidence/credible interval) and measures of statistical heterogeneity. If comparing groups, describe the direction of the effect.	Line 171-174
	20c	Present results of all investigations of possible causes of heterogeneity among study results.	Table 5
	20d	Present results of all sensitivity analyses conducted to assess the robustness of the synthesized results.	Table 6
Reporting biases	21	Present assessments of risk of bias due to missing results (arising from reporting biases) for each synthesis assessed.	Figure 3
Certainty of evidence	22	Present assessments of certainty (or confidence) in the body of evidence for each outcome assessed.	Line 174
**DISCUSSION**			
Discussion	23a	Provide a general interpretation of the results in the context of other evidence.	Line 188-191
	23b	Discuss any limitations of the evidence included in the review.	Line 192-215
	23c	Discuss any limitations of the review processes used.	Line 231-244
	23d	Discuss implications of the results for practice, policy, and future research.	Line 244-246

Section and Topic	Item #	Checklist item	Location where item is reported
**OTHER INFORMATION**			
Registration and protocol	24a	Provide registration information for the review, including register name and registration number, or state that the review was not registered.	N/A
	24b	Indicate where the review protocol can be accessed, or state that a protocol was not prepared.	N/A
	24c	Describe and explain any amendments to information provided at registration or in the protocol.	N/A
Support	25	Describe sources of financial or non-financial support for the review, and the role of the funders or sponsors in the review.	N/A
Competing interests	26	Declare any competing interests of review authors.	Line 250-252
Availability of data, code and other materials	27	Report which of the following are publicly available and where they can be found: template data collection forms; data extracted from included studies; data used for all analyses; analytic code; any other materials used in the review.	Supplementary Material

Supplementary Table S2 Data for dose response meta-analysis.

			range of bilirubin(μmol/L)						95%CI	
ID	Article	Type	Minimum	Maximum	Mean	Case	Total	HR/RR	Low	UP
1	1995-Breimer-before16:00	ci	1	7	4	90	788	1		
1	1995-Breimer-before16:00	ci	7	8	7.5	77	715	0.92	0.69	1.32
1	1995-Breimer-before16:00	ci	8	10	9	114	1371	0.68	0.51	0.89
1	1995-Breimer-before16:00	ci	10	12	11	79	905	0.75	0.55	1.03
1	1995-Breimer-before16:00	ci	12	86	49	110	1137	0.99	0.73	1.34
2	1995-Breimer-after16:00	ci	1	7	4	112	1150	1		
2	1995-Breimer-after16:00	ci	7	8	7.5	54	459	1.2	0.8	1.6
2	1995-Breimer-after16:00	ci	8	10	9	50	577	0.9	0.7	1.3
2	1995-Breimer-after16:00	ci	10	12	11	17	260	0.7	0.4	1.2
2	1995-Breimer-after16:00	ci	12	86	49	34	323	1.2	0.8	1.8
3	2010-Luc-man	ci	0.17	9.39	4.78	61	377	1		
3	2010-Luc-man	ci	9.4	11.1	10.25	21	285	0.41	0.25	0.68
3	2010-Luc-man	ci	11.11	14.52	12.815	48	576	0.55	0.37	0.8
3	2010-Luc-man	ci	14.53	17.94	16.235	30	380	0.57	0.37	0.89
3	2010-Luc-man	ci	17.95	66.69	42.32	36	365	0.75	0.49	1.14
4	2010-Luc-woman	ci	0.17	7.68	3.925	12	402	1		
4	2010-Luc-woman	ci	7.69	9.39	8.54	14	466	1.02	0.47	2.24
4	2010-Luc-woman	ci	9.4	11.1	10.25	10	418	0.64	0.27	1.52
4	2010-Luc-woman	ci	11.11	14.52	12.815	20	623	1.08	0.52	2.24
4	2010-Luc-woman	ci	14.53	46.17	30.35	5	384	0.53	0.18	1.52

5	2013-Stender	ci	0	9	4.5	623	17310	1		
5	2013-Stender	ci	10	12	11	466	12872	0.96	0.84	1.09
5	2013-Stender	ci	13	32.5	16.25	494	13526	0.93	0.82	1.06
6	2014-Jorgensen	ci	5	8	7	125	3032	1		
6	2014-Jorgensen	ci	8	10	9.5	85	2373	0.784	0.594	1.034
6	2014-Jorgensen	ci	10	13	11.9	69	2274	0.629	0.467	0.848
6	2014-Jorgensen	ci	13	25	17.7	76	2063	0.73	0.545	0.979
7	2018-Lai	ci	2.5	9.9	8.2	258	2412	1		
7	2018-Lai	ci	9.9	12	11	239	2534	0.89	0.74	1.06
7	2018-Lai	ci	12	14.1	13	196	2315	0.76	0.63	0.92
7	2018-Lai	ci	14.1	17.5	15.7	238	2422	0.9	0.75	1.07
7	2018-Lai	ci	17.5	59.7	20	273	2414	1.01	0.84	1.2
8	2018-Suh	ci	1.71	7.52	4.62	181	2951	1		
8	2018-Suh	ci	7.52	10.77	9.15	150	2942	0.916	0.727	1.154
8	2018-Suh	ci	10.77	34.2	22.49	113	2951	0.769	0.593	0.996
9	2018-Marconi	ci	0	6.84	3.42	564	24229	1		
9	2018-Marconi	ci	8.55	10.26	9.41	490	23641	0.85	0.76	0.96
9	2018-Marconi	ci	11.97	13.68	12.83	372	17463	0.89	0.76	1.04
9	2018-Marconi	ci	15.39	23.085	19.24	336	18524	0.81	0.7	0.93

ir：incidence rate, ci: Cohort study on cumulative incidence. RR: relative risk, HR: hazard risk, CI: confidence interval
